# Thiamine Deficiency in Hospitalized Veterans Without Alcohol Use Disorder: Prevalence, Biomarker Assessment, and Clinical Characteristics

**DOI:** 10.3390/diseases14080275

**Published:** 2026-07-31

**Authors:** Elisabeth A. Mates, Alexandrea Kilgore-Gomez, Claire Phibbs

**Affiliations:** 1 Department of Medicine, VA Sierra Nevada Healthcare System, Reno, NV 89502, USA; 2 School of Medicine, University of Nevada, Reno, NV 89557, USA; akilgoregomez@med.unr.edu; 3 Department of Research & Development, VA Sierra Nevada Healthcare System, Reno, NV 89502, USA; claire.phibbs@va.gov

**Keywords:** thiamine deficiency, thiamine deficiency disorders, thiamine-responsive disorders, beriberi, Wernicke encephalopathy, biomarker

## Abstract

Background: Thiamine deficiency (TD) causes thiamine deficiency disorders (TDDs) which are frequently overlooked in food-secure countries such as the United States (U.S.). Lack of awareness and the absence of validated, rapidly available biomarkers perpetuate underdiagnosis. Our objective was to determine prevalence of TD in hospitalized U.S. veterans without alcohol use disorder (AUD) and describe clinical characteristics of TDDs. A secondary objective was to evaluate the utility of plasma and whole-blood thiamine biomarkers in identifying thiamine-responsive disorders (TRDs). Methods: Newly hospitalized veterans without AUD were recruited. Fasting plasma and whole-blood thiamine were obtained. Interview, physical exam, and chart review were completed to assess clinical signs of TD. Participants were invited to return after thiamine repletion and changes in symptoms and exam findings were noted. Results: A total of 286 participants were enrolled: 261 had plasma thiamine results, 259 had whole-blood thiamine results, 179 completed interviews, 164 completed initial examination, and 60 returned for reexamination after repletion. A majority (86.59%) had potential signs or symptoms of TDDs, 26.44% had low plasma thiamine, 2.70% had low whole-blood thiamine, and 97.92% with low plasma thiamine had clinical signs of TDDs and many experienced multiple TD syndromes. Those with low plasma thiamine who returned after repletion showed improvements in cognitive scores (*p* = 0.0093) and motor strength (*p* = 0.0417), and most reported symptom improvement. Whole-blood thiamine missed many cases of TRDs. Conclusions: TD affects one quarter of hospitalized veterans without AUD. Low plasma thiamine identifies TRDs more frequently than low whole-blood thiamine. Using clinical criteria alone to diagnose TDDs overestimates cases and biomarker confirmation appears necessary.

## 1. Introduction

Thiamine is an essential micronutrient in humans and was the first B vitamin to be identified, hence the designation vitamin B1. Besides small (<2%) amounts of thiamine produced by gut microbiota, the body’s supply must be continuously ingested due to limited body stores (25–30 milligrams (mg)) [1,2]. Thiamine deficiency (TD) can develop quickly, in 2–3 weeks on a thiamine-deficient diet, or within 72 h in ill patients [3,4]. Thiamine is a cofactor in several key cellular energetic pathways and has non-cofactor roles in the nervous and immune systems [4,5]. When humans become thiamine-deficient, they develop a variety of clinical signs and symptoms historically described as “beriberi”, a Sinhalese word meaning extreme weakness, “I cannot, I cannot” [5]. More recently the phrase “thiamine deficiency disorders” (TDDs) was coined to describe the observation that individuals with TD often have symptoms of multiple beriberi syndromes concomitantly [5,6]. TD is thought to be rare in high-income countries, especially where thiamine fortification of food staples is routine, although prevalence data are lacking [6]. The exception is those with alcohol use disorder (AUD) because of frequent concomitant malnutrition and because alcohol impairs the intestinal absorption of thiamine [6]. Left untreated, TD is fatal [6,7] and often undiagnosed prior to death, particularly in populations that do not have obvious risk factors [7]. It is unknown what the best method is to diagnose TDDs, or which biomarker is best suited to confirm diagnosis.

Ingested thiamine is absorbed in the upper small intestine by thiamine transport proteins at low concentrations, and by passive diffusion at high intestinal concentrations [2,4,8]. After passing through enterocytes in the gut, it is released into plasma, circulated, and taken up by cells. In whole blood, most of the thiamine exists as thiamine diphosphate (TDP) in red blood cells, while plasma contains free thiamine and thiamine monophosphate (TMP). Once in the cell cytosol, free thiamine can be phosphorylated into four forms: TMP, which is transported across cell membranes [3]; TDP, the most prevalent form of thiamine in the body, which has cofactor and non-cofactor roles in the cytosol and mitochondria [3,4,9]; thiamine triphosphate (ThTP), which plays non-cofactor roles in membrane conductance and is a phosphate donor in energy metabolism [4]; and adenylated ThTP, the function of which is being investigated [4]. TDP is a cofactor for four intracellular bioenergetic enzyme systems (three in mitochondria, one in cytosol) that require continuous supply [4]. They include the pyruvate dehydrogenase complex (PDC), the α-ketoglutarate dehydrogenase complex (α-KGDHC), transketolase in the pentose phosphate pathway, and the branched chain α-ketoacid dehydrogenase complex (BCKDH) [4]. In states of TD, there is reduced activity of these bioenergetic pathways, which results in the production and accumulation of lactate and free radicals which can have toxic effects, particularly in the brain [4]. Thiamine and its phosphate esters have non-cofactor roles in the central nervous system as an active component of the axoplasmic, mitochondrial, and synaptosomal membranes [4]. It is involved in cellular differentiation, synapse formation, axonal growth, and myelinogenesis [4]. It participates in the maintenance of the nerve membrane and supports the synthesis of myelin and several neurotransmitters: acetylcholine, serotonin, and amino acids [4]. It inhibits the activity of acetylcholinesterase which breaks down acetylcholine, a key neurotransmitter [4]. When deficiency occurs, evidence suggests tissues are affected at different rates, including the various cellular enzyme systems that utilize TDP. Even different regions of the same organ such as the brain can be affected at varying rates [10]. Available biomarkers of thiamine are typically measured in blood (the most available biomatrix) but may not reflect target tissue concentrations in states of deficiency [11].

In adults, clinical expression of TD likely depends on genetic susceptibility, concomitant disease, age, and other factors [12]. Historically syndromes of TD were described by four distinct clinical entities. Wet beriberi is heart failure characterized by pulmonary edema, shortness of breath, heart palpitations, peripheral edema, hypotension, lactic acidosis, or tachycardia [12,13]. Dry beriberi is peripheral polyneuropathy with some mixture of weakness, muscle pain, neuropathy, or loss of tendon reflexes, typically worse distally, and can mimic Guillain Barré syndrome [12,14]. Gastrointestinal (GI) beriberi describes a constellation of symptoms including anorexia, nausea, dysphagia, vomiting, constipation, abdominal pain, or lactic acidosis [15]. Wernicke’s encephalopathy (WE) is brain dysfunction characterized by some combination of altered mental status (confusion, delirium, memory impairment, etc.), ophthalmoplegia, and ataxia [7,12]. Left untreated, WE can progress to coma and death [7,12]. There are many who think alcohol-related and alcohol-unrelated WE have different clinical expressions, making alcohol-unrelated WE harder to diagnose [16,17,18]. Mental health symptoms such as depression, agitation, personality change, hallucinations, and seizures can also occur in TD, probably due to both the cofactor and non-cofactor roles thiamine plays in the central nervous system [4,12].

In an ideal world, the diagnosis of TDDs would be based on clinical signs and symptoms that improve with thiamine replenishment (thiamine-responsive disorders (TRDs)), supported by confirmatory biomarker levels [19,20]. In practice, diagnosis is challenging due to non-specific signs and symptoms and the lack of a rapidly available gold standard biomarker with agreed-upon reference intervals [9,20]. The original biomarker technique, the erythrocyte transketolase activation assay (ETKA), measures the functional capacity of the TDP-dependent enzyme transketolase in erythrocytes [9]. The advantages of ETKA are that it is a functional measurement of TD and fasting samples are not required [6,21]. The disadvantages are many [6,9,21,22], chiefly, that it is no longer available in commercial laboratories. The most widely available commercial test is high-performance liquid chromatography (HPLC) coupled with mass spectroscopy (MS) which can be used to measure plasma, whole-blood, or washed red blood cell (RBC) thiamine levels [9,21]. The disadvantages include a lack certified reference materials or methods to ensure comparability of results from different laboratories [9]; there are no gold standard biomatrices or reference ranges that reflect TRDs with certainty [6,9]; and samples must be carefully handled and shipped to reference laboratories with a 1–2-week delay in obtaining results [6]. Some recommend measurement of TDP in RBCs or whole blood as the method that best reflects thiamine body stores [6,23]. Measurement of plasma thiamine, a combination of free thiamine and TMP, has been criticized because they make up a small proportion of the total thiamine in the body (TDP being the most prevalent) and reflect recent intake rather than being an indicator of tissue thiamine status [6]. In essence, there is no readily available diagnostic paradigm to assist clinicians in diagnosing TDDs (especially when risk factors are not obvious), a critical deficiency perpetuating underdiagnosis and treatment.

There are many steps required to validate a biomarker of disease [24]. In addition to laboratory techniques being reproducible and accurate, a vitamin biomarker should correlate with clinical features of deficiency to ensure its usefulness [25,26]. Collie et al. discuss technical concerns with the most widely available thiamine biomarkers, which we will not repeat here [9]. One common source of error in the use of biomarkers to diagnose disease is that reference ranges are often determined using healthy volunteers (with no guarantee of age, sex, or racial diversity) whereas their clinical utility lies in detecting abnormal states [25]. One method of validation of a biomarker is by prospective clinical investigations where use of the biomarker to guide patient care improves health outcomes [26]. Hess et al. recently completed an investigation of ETKA and whole-blood thiamine in assisting with the diagnosis of TD in infants and young children in Laos and found neither were able to distinguish TRDs reliably [27]. This emphasizes a persistent knowledge gap regarding which biomarker of TD is best suited to verify the diagnosis of TRDs.

The objective of this prospective study was to fill the knowledge gap of the prevalence of TDDs in hospitalized U.S. veterans without AUD using both clinical and biomarker data. This report expands on preliminary data presented in a previous publication [28] by incorporating data from more individuals and presenting detailed description of TD symptoms, syndromes, and syndrome overlap. A secondary objective was to determine whether plasma thiamine, whole-blood thiamine, clinical criteria or some combination of these are best suited to identify cases of TRDs.

## 2. Materials and Methods

### Ethics Statement

These studies were approved by the University of Nevada, Reno Institutional Review Board (full study #1867369, truncated study #1899906). We have adhered to the ethical principles of the Declaration of Helsinki. Written informed consent was obtained from each participant. The full study was registered with ClinicalTrials.gov: NCT05480943.

This is a prospective prevalence pilot study of thiamine deficiency in adult veterans admitted to the VA Sierra Nevada Healthcare System (VASNHCS) hospital. Portions of the study protocol were previously described [28]. All new admissions to the hospital were screened and veterans without excess alcohol intake were approached for participation in the study. Exclusion criteria included B-complex vitamin or thiamine use prior to admission, excess alcohol consumption (>14 drinks per week for men less than 65 years old, or >7 per week for men 65 years or older or women), inability to comprehend and “read back” basic elements of the informed consent (as determined by the U-ARE protocol [29], see Appendix A), or living more than 70 miles from the hospital (a barrier to follow-up) [28].

We administered survey questions to determine if participants had signs or symptoms of TD (Appendix B). Notably, we did not include wet beriberi symptoms of shortness of breath or leg edema due to a high percentage of participants with chronic lung and heart disease. We inquired about appetite, recent food intake, and recent weight loss (Appendix B). We assessed tobacco, alcohol, and recreational drug use and inquired about vitamin and over-the-counter drug or supplement use.

Early morning fasting blood was drawn for measurement of prealbumin, plasma, and whole-blood thiamine. Plasma thiamine was analyzed by Quest Diagnostics Valencia, CA, USA using liquid chromatography/mass spectroscopy (LC-MS) [30] with a normal range of 8–30 nanomoles/liter (nmol L^−1^). Whole-blood thiamine was analyzed at LabCorp Burlington, NC, USA using LC-MS [30] with a normal range of 66.5–200.0 nmol L^−1^. Standardized physical exams were carried out late in the hospital stay after the medical condition for which they were hospitalized [28]. We assessed extraocular movements, motor strength, peripheral sensation, cerebellar function, peripheral edema, physical signs of malnutrition, and used auscultation to assess heart and lung sounds [28]. Mental status as assessed using the Montreal Cognitive Assessment (MOCA, www.mocacognition.com, URL accessed on 13 October 2022) with a score of 25 or less considered abnormal (17 or less for those with vision impairment after eliminating visual portions). Malnutrition was determined using GLIM criteria [31].

After interview and examination, it was determined whether an individual met criteria for a TD clinical syndrome: WE, wet beriberi, dry beriberi, or GI beriberi [28]. Initially, we determined whether they had WE using the Caine criteria [32] where thiamine deficiency was suspected if they met criteria for malnutrition [31]. Once biomarker results were available, we reevaluated whether they had WE if they had low plasma thiamine, which was considered sufficient evidence of dietary deficiency of thiamine. Our determination of wet beriberi was based on the presence of pulmonary rales or peripheral edema with no plausible alternate explanation [28]. Our determination of dry beriberi was based on the presence of motor weakness in more than 2 muscle groups or acute onset peripheral neuropathy not explained by other conditions [28]. Our determination of GI beriberi was based on presence of symptoms of anorexia, nausea, vomiting, or constipation in the absence of alternative explanation [28].

If they met clinical criteria for WE, we advised the inpatient treatment team to administer high-dose IV thiamine every 8 h and we prescribed oral thiamine 100 mg daily for 2 weeks on discharge. For participants without WE who had signs or symptoms of other syndromes or had low thiamine levels, we prescribed oral thiamine 100 mg daily for 2 weeks at discharge. Those with TD clinical syndromes or low thiamine levels were invited back for reevaluation. We verified they had taken the tablets and repeated thiamine biomarker measurements. We inquired whether previous symptoms improved and reexamined them using the same standardized exam. Composite scores were calculated for extraocular movements (sum of normal = 1, nystagmus or disconjugate gaze = 0 for each of 7 gaze directions) and motor strength (sum of 0 to 5 for 10 muscle groups), and ataxia (normal = 1, ataxia = 0 for each of 5 tests), and compared before and after thiamine repletion. If an element of an exam was untestable on one visit, it was removed from the other visit to ensure scores were comparable.

In a separately funded smaller investigation, study procedures were truncated to include only biomarker evidence of TD. In these individuals, we did not exclude them based on their admission status to the hospital or distance lived from the medical center. Fasting plasma and whole-blood thiamine were obtained. We did not administer surveys or conduct physical exams. For both studies, we performed medical record reviews for demographics, hospital length of stay (LOS), readmissions, and death [28].

All measurements were taken and reported once for each participant. The correlation between plasma thiamine and whole-blood thiamine was calculated using both Pearson and Spearman rank correlation. The prevalence of low plasma thiamine and low whole-blood thiamine was calculated as patients with low thiamine biomarker levels divided by patients in the cohort with thiamine biomarker results. Measures of association between categorical and binary variables (e.g., demographic features, readmission from prior 30-day hospitalization, death within 90 days) were assessed using Pearson chi-squared test *p*-values, as well as respective odds ratios (ORs) and 95% confidence intervals (CIs) from univariate logistic regressions. If 25% of cells had expected counts of less than five, Fisher’s exact test replaced Pearson chi-square to determine statistical significance. Measures of association between continuous variables (e.g., LOS) were assessed using Wilcoxon rank-sum and negative binomial regression, adjusted for age and birth sex as covariates due to overdispersion and non-normal distribution of LOS (Shapiro–Wilk *p* > 0.05). Statistical significance was determined by a two-sided *p*-value < 0.05. Participants with missing values for a given variable were excluded from statistical analyses because they were <10% of the sample. Missing values were retained and reported descriptively as “unknown” when applicable. To assess the differences in physical examination finding scores (MOCA, extraocular movements, motor strength, and ataxia) before and after treatment with thiamine repletion, two-sided t-tests and two-sided Wilcoxon signed rank tests were utilized. T-tests were used for normally distributed MOCA scores, while Wilcoxon signed-rank tests were used for non-normally distributed physical examination scores. Distribution was assessed with Shapiro–Wilk *p*-values < 0.05. All analyses were conducted using RStudio 2024.09 and R 4.4.1 on the Veterans Affairs Informatics and Computing Infrastructure (VINCI) Austin, TX, USA and Microsoft Excel for Microsoft 365 MSO, version 2408, Seattle, WA USA.

## 3. Results

### 3.1. Participants

**Figure 1:**
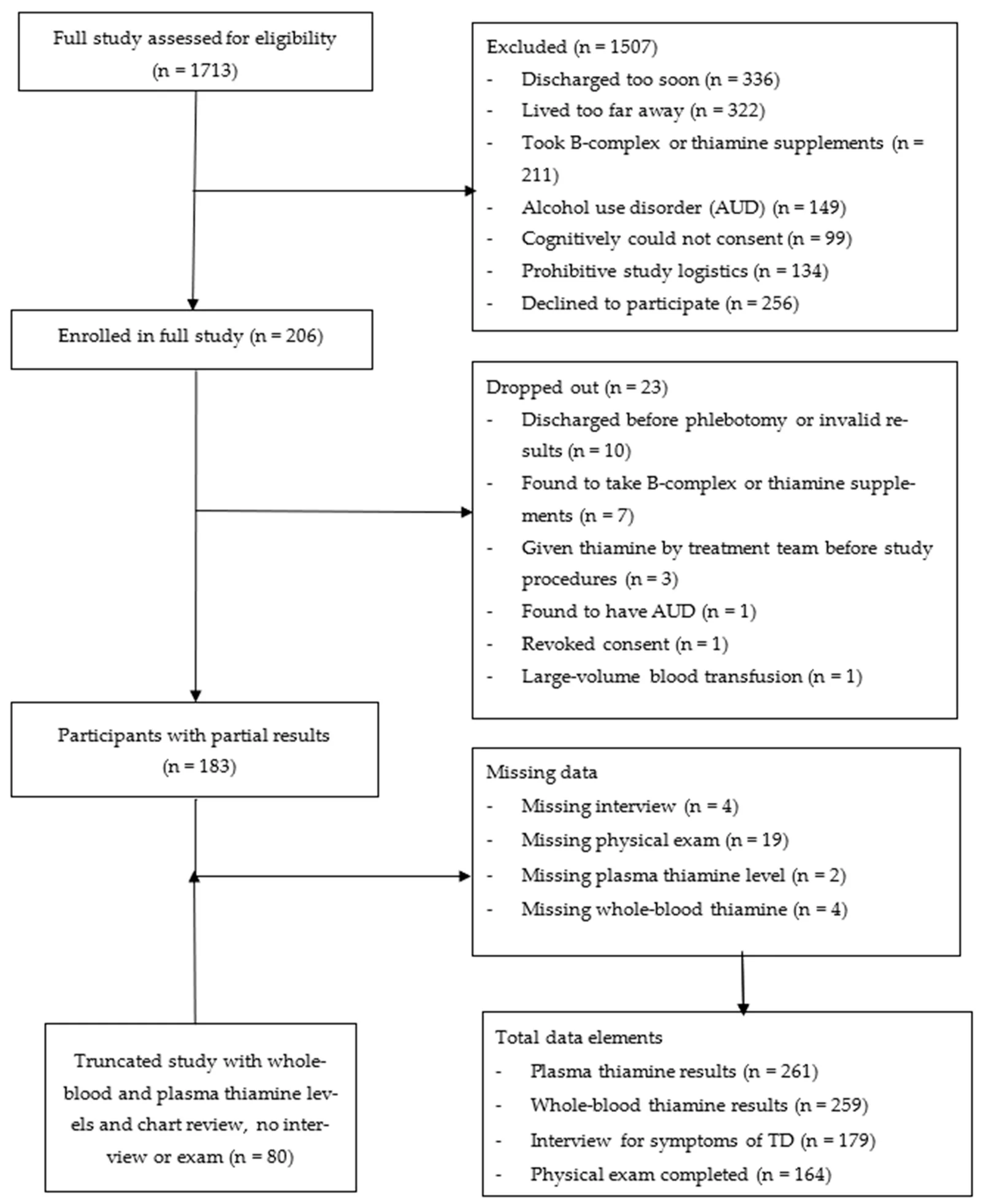
Flowchart of participants.

Figure 1 shows the flow of participants in both the full and truncated studies. A total of 206 participants were enrolled in the full study: 183 had partial, and 155 had complete results (exam, interview, and both biomarker results). Screening data for the truncated study were incomplete and 80 were enrolled, totaling 286 enrolled participants. Of those, there were 261 with plasma thiamine results, 259 with whole-blood thiamine results, 179 who completed interviews, and 164 who completed physical examinations. A total of 60 returned for reevaluation after thiamine repletion.

### 3.2. Prevalence of Thiamine Deficiency Biomarkers

Table 1 shows characteristics of participants versus prevalence of plasma thiamine and whole-blood thiamine deficiency for 263 participants. The average age was 70.64 years, and the majority were white non-Hispanic males, reflective of our inpatient population. There were 69 (26.44%) with low plasma thiamine and seven (2.70%) with low whole-blood thiamine levels. There were no statistically significant associations among low plasma thiamine (deficient vs. not deficient) and age, birth sex, ethnicity, or race. There were no statistically significant associations between low whole-blood thiamine and ethnicity or race. There were significant associations among low whole-blood thiamine, age (*p* = 0.0340) and birth sex (*p* = 0.0255).

### 3.3. Correlation Between Plasma and Whole-Blood Thiamine Biomarkers

**Figure 2:**
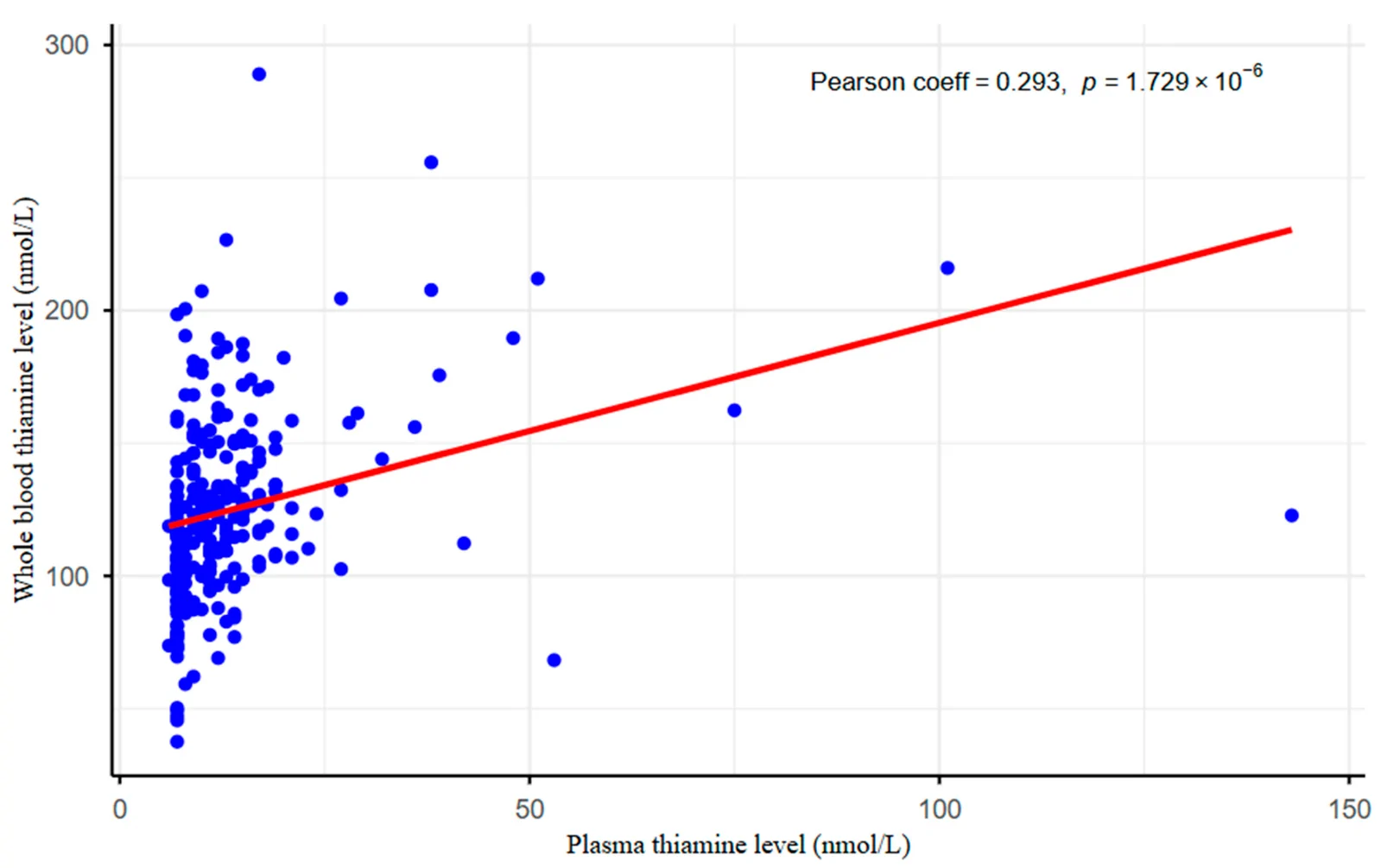
Plot of plasma thiamine vs. whole-blood thiamine levels with linear correlation coefficient of 0.293.

**Figure 3:**
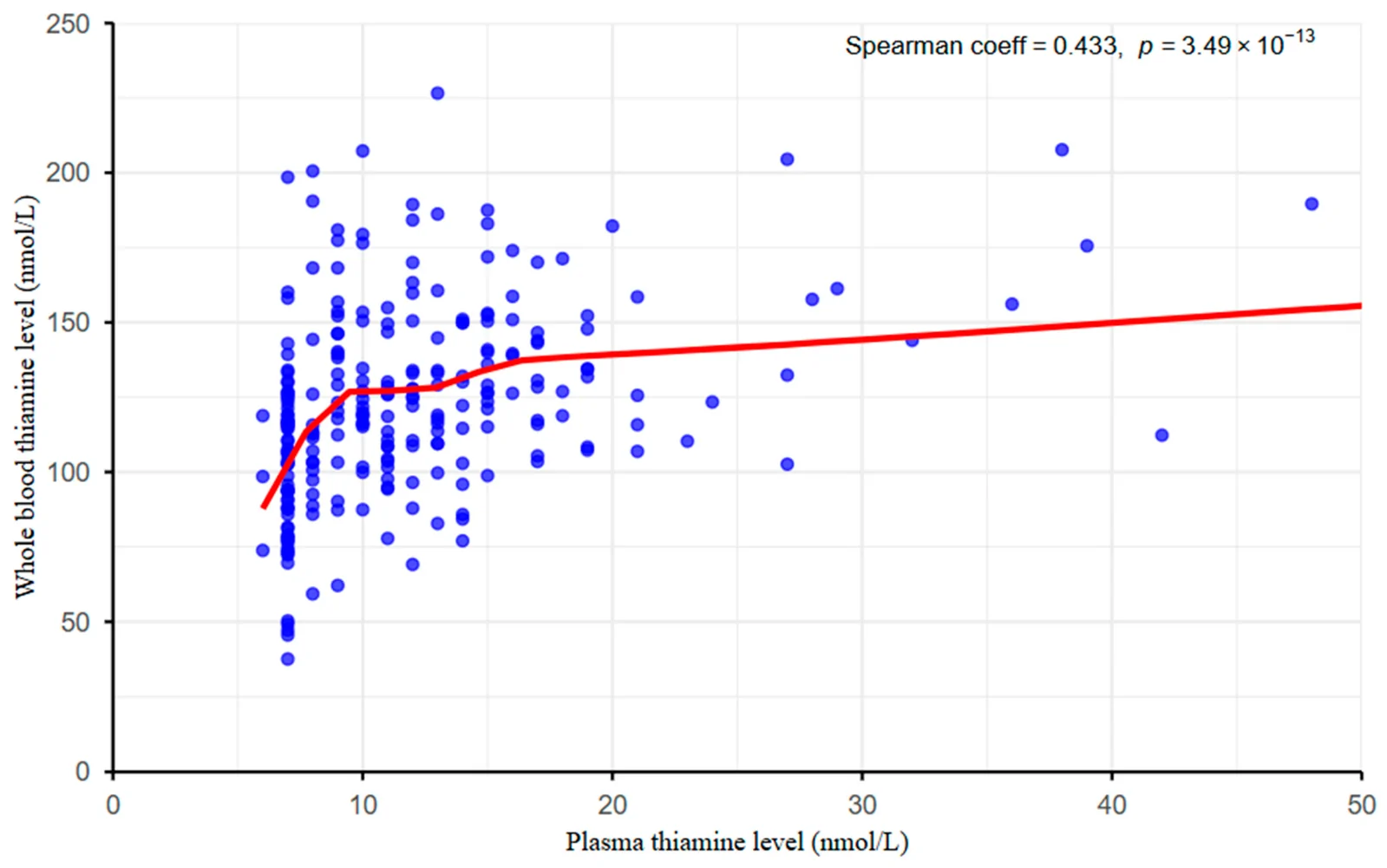
Plot of plasma thiamine vs. whole-blood thiamine levels with nonlinear correlation coefficient of 0.433.

We looked at the correlation between simultaneously drawn plasma thiamine and whole-blood thiamine. Pearson correlation analysis showed a significant but weak linear association (*p* < 0.0001, r = 0.293) (Figure 2). Spearman correlation analysis demonstrated a moderate monotonic non-linear relationship (*p* < 0.0001, r = 0.433) (Figure 3).

### 3.4. Prevalence of Symptoms and Syndromes of Thiamine Deficiency

Of 164 participants in the full study who completed interview and examination, 142 (86.59%) met clinical criteria for a TD syndrome. Of the remaining with no TD syndromes, four had low plasma thiamine and none had low whole-blood thiamine. Of those meeting criteria for a TD syndrome, 99 (60.37%) met criteria for WE, 92 (56.10%) met criteria for dry beriberi, and 12 (7.32%) met criteria for wet beriberi. Of 179 who completed interviews, 95 (53.07%) met criteria for GI beriberi and 173 (96.65%) had at least one symptom of TD.

**Figure 4:**
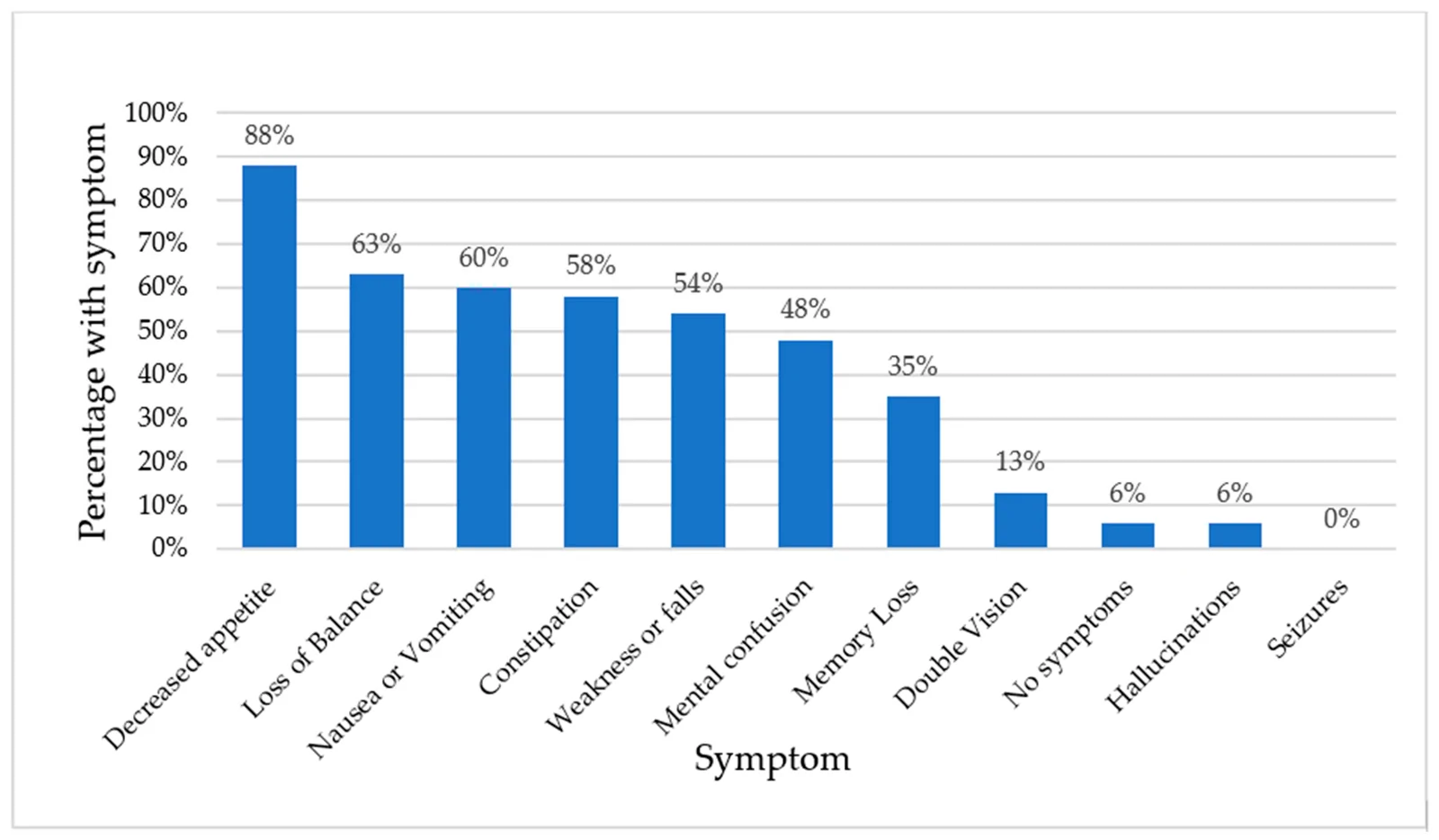
Symptoms associated with low plasma thiamine (<8 nmol L^−1^) for 48 participants.

Of 48 cases of low plasma thiamine where clinical data were collected, five were missing exams. All but one (97.92%) had symptoms and/or physical signs of a TDD. Figure 4 shows the frequency of symptoms of TD for those with low plasma thiamine. The average symptom count was 4.25 with the range 0 to 8. Decreased appetite was the most frequently reported symptom with no one reporting seizures. Three individuals reported no symptoms, two of whom had physical findings of a syndrome.

**Figure 5:**
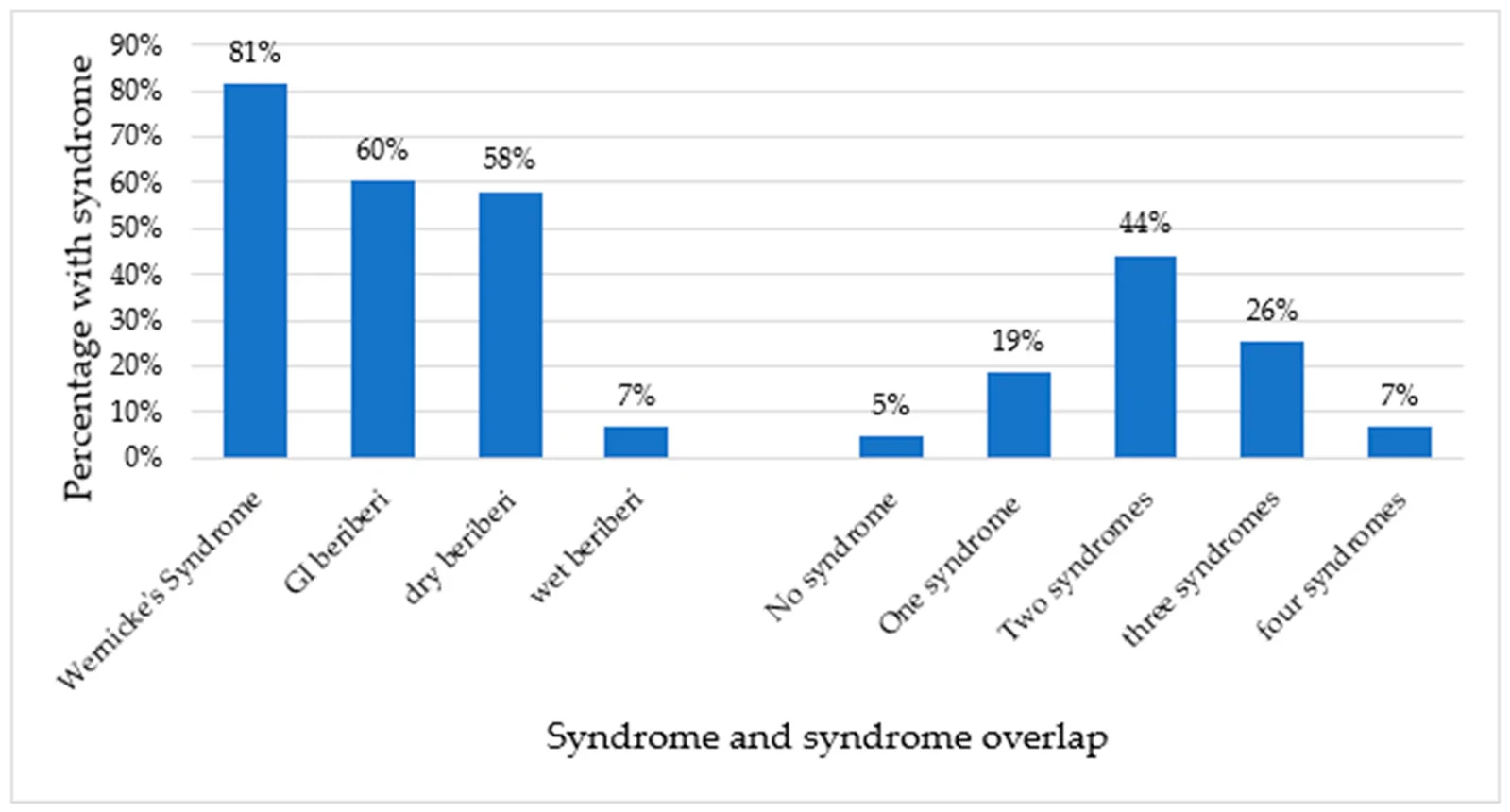
Thiamine deficiency syndromes associated with low plasma thiamine (<8 nmol L^−1^) for 48 participants.

Of five cases of low whole-blood thiamine where clinical data were collected, all completed exams. All had symptoms and physical signs of a TD syndrome. All had decreased appetite, four of five had loss of balance, nausea or vomiting, and weakness. Two complained of mental confusion, one constipation, one memory loss, and one reported hallucinations. None complained of double vision or seizures. Of the four clinical syndromes of TD, GI beriberi was based on symptoms (N = 48), and the remainder were based on exam findings (N = 43). Figure 5 shows the frequency of clinical syndromes and syndrome overlap in those with low plasma thiamine. WE was the most common syndrome with 35 of 43 having it, followed by GI beriberi (29 of 48), dry beriberi (25 of 43), and wet beriberi (3 of 43). Four did not meet criteria for any syndrome and eight had one, 19 had two, 11 had three, and three had all four syndromes.

Of five participants with whole-blood thiamine deficiency, all had WE, three had GI beriberi, three had dry beriberi, and none had wet beriberi. One had only one syndrome, two had two syndromes, two had three syndromes, and none had all four syndromes.

**Figure 6:**
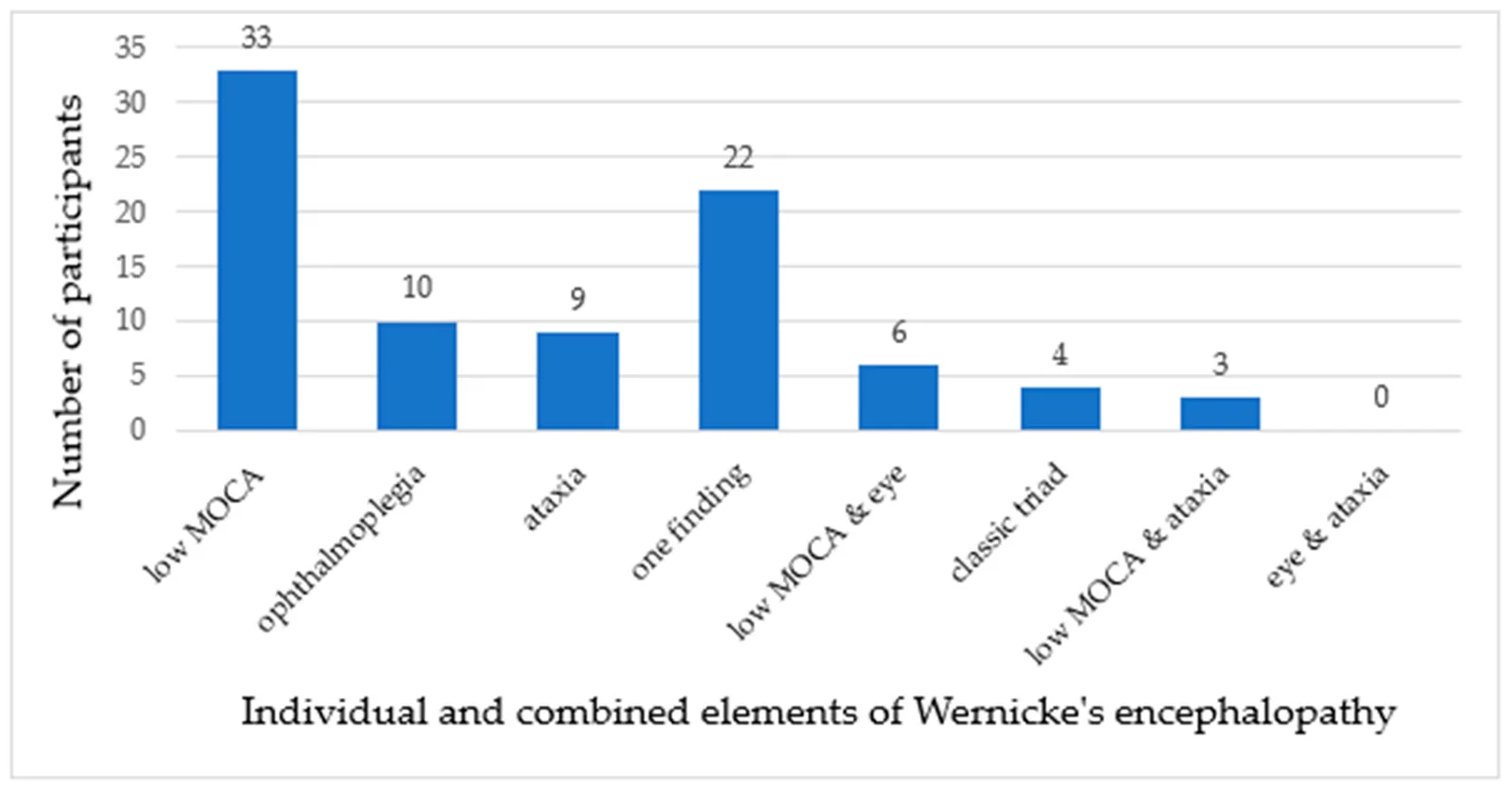
Elements of Wernicke’s encephalopathy for 35 participants with low plasma thiamine (<8 nmol L^−1^) who met criteria.

Thirty-five participants with low plasma thiamine had WE using the Caine criteria where nutritional deficiency was defined as low plasma thiamine, and they had at least one of the three findings: encephalopathy (measured by low MOCA), ophthalmoplegia, or ataxia. A total of 33 of 35 had low MOCA scores, 10 of 35 had ophthalmoplegia, and nine of 35 had ataxia (Figure 6). The majority had only one clinical finding of WE, and four of 35 had the classic triad.

### 3.5. Clinical Improvement After Thiamine Replenishment

Of 164 participants where physical examination was completed, 60 returned for reexamination of which 32 had low plasma thiamine and two had low whole-blood and low plasma thiamine. Table 2 shows a comparison of those who followed up and those who did not. There was a statistically significant difference in the age distribution of those with follow-up (*p* = 0.0456) but not the average age (*p* = 0.2000). The average plasma thiamine was lower in the follow-up group (*p* = <0.0010) as well as the percentage with plasma thiamine less than 8 nmol L^−1^ (*p* = <0.0010). There was a significant difference in the average symptom count with more symptoms in those who followed up than did not (*p* = 0.0140).

Of those with low plasma thiamine, 27 of 32 reported symptom improvement, four of 32 were asymptomatic, and one of 32 reported no symptom improvement at follow-up. There was a statistically significant improvement in MOCA test scores by an average of 1.9 points (*p* = 0.0093) and composite motor strength by an average of 2.5 points (*p* = 0.0417) (Table 3). Extraocular movement and ataxia composite scores displayed a median improvement but were not statistically significant. Repeat plasma thiamine testing showed that 24 of 32 were no longer deficient, one was still deficient, and seven did not complete lab reevaluation but reported compliance with thiamine tablets. For the two with low whole-blood thiamine, one had improvement in both clinical and symptom scores, while the other showed no improvement in either.

Table 4 shows associations between low plasma and whole-blood thiamine and measures of hospital outcomes for both the full and truncated studies for 263 participants. As with our prior publication [28], we did not find a statistically significant increase in readmission rates, death within 90 days, or longer LOS with a larger sample size. We completed a subgroup analysis of these metrics for individuals with WE and each form of beriberi and did not find significant associations.

We evaluated the association between low thiamine biomarkers and GLIM malnutrition and did not find a statistically significant association between low plasma thiamine (*p* = 0.2149, O.R. 1.557, 95% C.I. [0.775–3.175]) or low whole-blood thiamine (*p* = 0.0591, O.R. 11.137, 95% C.I. [1.230–14,790.9]) and malnutrition.

We evaluated whether low plasma or whole-blood thiamine was correlated with smoking or recreational drug use. For smoking we used data from both the full and truncated studies (263 participants) and for recreational drug use we used data from the full study where we verified use (164 participants). We did not find a significant relationship between smoking and low plasma thiamine (*p* = 0.1989, O.R. 1.503, 95% C.I. [0.795–2.786]) or low whole-blood thiamine (*p* = 0.6599, O.R. 1.368, 95% C.I. [0.192–6.535]). Similarly, we did not find significant relationships between recreational drug use and low plasma thiamine (*p* = 0.2852, O.R. 1.595, 95% C.I. [0.655–3.719]) or low whole-blood thiamine (*p* = 1.0000, O.R. 1.152, 95% C.I. [0.058–8.159]).

## 4. Discussion

This report expands on our prior publication [28] and is the first prevalence study of TD in hospitalized adults without AUD in a high-income country, addressing several knowledge gaps. We found that the prevalence of TD as determined by low plasma thiamine was 26.44%, whereas the prevalence of low whole-blood thiamine was much lower at 2.70%. It should be emphasized that there is no consensus on reference intervals for thiamine biomarker results and we used reference ranges provided by commercial laboratories to determine deficiency [9]. We found that those with low plasma thiamine levels had associated signs and symptoms of TDDs with only one individual being asymptomatic, suggesting that the cutoff levels identified clinically relevant deficiency. Most had multiple concurrent TD syndromes, highlighting the fact that TD causes multisystem dysfunction. For 32 individuals with low plasma thiamine who attended follow-up appointments, there were significant improvements in TD symptoms, cognition, and strength after thiamine replenishment. This suggests cases identified by low plasma thiamine benefit from thiamine repletion with improved health outcomes, which is key to validating it as a useful biomarker. Low whole-blood thiamine was not a sensitive biomarker of TRDs, missing many cases that improved with repletion. Clinical signs and symptoms of TDDs were present in most enrollees, which overestimate cases when used without biomarker confirmation.

Of those with low plasma thiamine who returned for reevaluation after repletion, most showed improvement in both symptoms and physical exam findings. While some of these improvements can be attributed to recovery from the acute illness for which they were hospitalized, we attempted to mitigate this by examining individuals late in their hospital stay. Those who followed up had lower average thiamine levels, higher symptom counts, and were predominantly thiamine-deficient compared to those who did not follow up. We only invited those with low thiamine levels or symptomatic individuals to follow up due to funding limitations, introducing selection bias. Future studies should include larger numbers of participants, all of whom are invited to follow up to eliminate such bias.

Relatively few individuals had low whole-blood thiamine, limiting our ability to evaluate statistical associations with demographics, clinical characteristics, and hospital metrics. The significant associations with age and birth sex should be interpreted with caution given the low numbers with abnormal results. The discrepancy between whole-blood thiamine deficiency and symptomatic plasma thiamine deficiency suggests whole-blood thiamine lacks sensitivity to TRDs. This contrasts with the expert opinion that whole-blood thiamine best represents total body stores of thiamine [6,23]. The Spearman correlation suggests partial agreement between whole-blood thiamine and plasma thiamine, while raising the possibility that whole-blood thiamine is delayed or less responsive to change in thiamine status compared to plasma thiamine. As we previously discussed, we believe this is due to differences in cellular bioenergetics that consume TDP in RBCs (which lack nuclei and mitochondria) rather than nucleated cells elsewhere in the body [33]. This is supported by results from Warnock et al. who demonstrated that the rate of depletion of TDP in RBCs in rats on a thiamine-deficient diet was 5–6 days slower than that in liver, heart, and brain [11]. In addition to our study, several others found that low whole-blood thiamine missed cases of TDDs [27,34]. These studies, combined with our results, suggest that whole-blood thiamine is insensitive to TRDs using the currently established cut points, limiting its clinical utility, a key requirement for biomarker validation [24]. Future studies with more diverse individuals are required to fully evaluate the utility of whole-blood thiamine as a biomarker of TRDs.

Our data indicates that using clinical criteria without biomarker confirmation to make a diagnosis of TD produces excess false positive results. More than 85% of our participants had symptoms or met criteria for a TDD, far more than can reasonably be expected. Using low plasma thiamine together with clinical signs identified TRDs in our study, although further evaluation to determine appropriate reference ranges is needed. Except for WE, beriberi syndromes do not have agreed-upon diagnostic criteria. Symptoms of TD are non-specific and suffered by many adults without TD, making diagnosis solely on clinical grounds too difficult. Even though diagnostic criteria exist for WE, there are many potential explanations for abnormalities in each component. For example, abnormal results for the MOCA (or similar cognitive test) can occur from drug side effects, abnormal oxygenation, hypercarbia, renal failure, and a plethora of other reasons. In this study, we used liberal criteria for diagnosing wet, dry, and GI beriberi, such that if any symptoms were present and not explained by concomitant illness, they were classified as having the syndrome. Diagnosing TD based on clinical signs or symptoms without biomarker confirmation would lead to many false positives.

Many argue that the components of plasma thiamine (free thiamine and TMP) are not the biologically active forms of thiamine and represent recent intake rather than target tissue levels [6]. Despite this, our exploratory results indicate that it may have clinical utility in diagnosing TRDs when combined with clinical findings. Given that plasma is the compartment from which thiamine is transported into tissues, low values suggest there is little available for uptake by potentially deficient organs. On the other hand, low plasma thiamine does not necessarily mean low tissue thiamine levels, and some evidence shows that it drops more quickly than tissue levels on deficient diets [35]. If this is the case, the use of plasma thiamine in a diagnostic paradigm would overestimate cases of TD. The risk of such an overly sensitive biomarker is excess false positives leading to inappropriate treatment. The risk of an insensitive but specific biomarker is excess false negatives and untreated disease. Thiamine replenishment is inexpensive and has an excellent safety profile, making treatment low risk [36]. On the other hand, a missed diagnosis of WE (which happens frequently [7,23]) can lead to Korsakoff dementia or death. Our study suggests that low plasma thiamine combined with clinical signs identifies potential cases of TD that when given thiamine repletion, may lead to better health outcomes, a requirement of a good biomarker.

In the real world of patient care, a useful biomarker needs to be available in a timely manner, especially when treatment delays risk irreversible tissue damage as they do in WE [37]. One drawback to using plasma thiamine in a diagnostic paradigm is the delay in obtaining results. Dependence on HPLC-MS technology means that frozen specimens must be shipped to reference laboratories, which for us resulted in a 7- to 10-day delay in obtaining results (which could be longer in more remote areas). Currently, the solution to this barrier is empirical treatment while waiting for results. In practice, there is hesitation to commit to therapy when a diagnosis is uncertain, especially if clinical expression of a disease is non-specific as it is with TDDs. A rapid point-of-care biomarker is desperately needed to fill this gap. The technique described by Edwards et al. is a promising candidate, and future studies of TRDs could be used to validate it [38].

Several authors have postulated that clinical findings of WE differ in those without AUD, with fewer having ophthalmoplegia and ataxia, making it more difficult to recognize [7,16,17,18]. In this study we found that the most common manifestation of WE was altered mental status (abnormal MOCA) at 94% with only 29% having ophthalmoplegia, 26% having ataxia, and 11% having the triad. This was surprisingly similar to cases of pathologically confirmed WE in those with AUD described by Harper et al. in 1986 [39]. Several other studies found differences, though. Galvin et al. compared 102 AUD-related WE cases to 116 cases unrelated to AUD [7]. They concluded that ophthalmoplegia and ataxia were more frequent among those with AUD. Chamorro et al. completed a similar comparison of 464 cases with AUD to 34 without AUD. They found that those with AUD more frequently had ataxia and less frequently had ophthalmoplegia [18]. Zuccoli et al. compared clinical and MRI findings between the two groups for 56 cases (24 with AUD, 32 without) [40] and found that those with AUD more often had ophthalmoplegia and ataxia. In the studies described by Galvin, Chamorro, and Zuccoli, the percentage with ophthalmoplegia and ataxia were 50% or more in both groups, more prevalent than ours and Harper’s findings. The low incidence of ophthalmoplegia, ataxia, and the classic triad in non-alcohol users would make WE harder to recognize, especially in the absence of confirmatory biomarker results.

We evaluated whether TDDs are associated with a higher risk of hospital readmission after discharge, longer LOS, or increase in 90-day mortality [41,42]. Most participants with TDDs had multiple concurrent TD syndromes affecting several organ systems, which in theory would increase some of these metrics. Despite the addition of 80 cases to our previous analysis [28], we did not observe any differences in these metrics between those with TDDs and those without them. It is likely that our low enrollment limited our ability to control for confounding factors, and the fact that our study was not powered to detect these effects explains this finding.

The lack of association between malnutrition and TD was surprising, although our study was not powered to detect this. The use of GLIM criteria without concomitant assessment by a dietician may have led to misdiagnosis of malnutrition. Another possibility is that thiamine is heat-labile and cooking breaks down 20–70% of the vitamin [1,4]. Causes of TD can be due to insufficient intake (low intake, malabsorption, or toxin interference), increased consumption of thiamine stores (in critical illness), and increased losses (due to vomiting, diarrhea, dialysis, or chronic diuretic use) [43]. It is possible that acute or chronic illnesses or medication use led to TD from one of these mechanisms in the absence of malnutrition. If future studies confirm a lack of association between TD and malnutrition, identifying cases of TD could become even more difficult.

Limitations of this study include the exploratory pilot design, small sample size, and a non-diverse single-center population. Enrollment also fell short of the intended goal, so the study was underpowered, limiting our ability to provide generalizable results and control for potential confounders. We were unable to simultaneously evaluate ETKA as a biomarker of TD due to lack of access to this test. We did not complete food intake surveys to determine whether dietary inadequacies contributed to cases of TD due to funding and resource limitations. Our chosen clinical criteria for diagnosis of wet, dry, and GI beriberi were potentially too liberal, owing to a lack of diagnostic criteria and the non-specific signs and symptoms for each syndrome. The use of the MOCA test to assess encephalopathy in the diagnosis of WE is similarly non-specific, possibly leading to overdiagnosis. There were confounding factors not accounted for including but not limited to socioeconomic status, concomitant medical illness, and medication use. Improvements in mental status and physical functioning could have been due to resolution of acute medical problems treated in the hospital rather than replenishment of thiamine. Complete separation was present for many of the associations tested due to small cell counts, resulting in unstable estimates and limited reliability of results. Significant associations should therefore be interpreted with caution and warrant further investigation.

Future studies should include larger, more diverse patient populations with nutritional assessments by registered dieticians. Further, analysis of appropriate cutoff values for plasma and whole-blood thiamine using TRDs would be helpful. The investigation of comorbid medical conditions and medications to identify potential risk factors for TD would be valuable.

## 5. Conclusions

Clinically evident TDDs affect about one quarter of hospitalized veterans without AUD. Using clinical criteria alone to diagnose TDDs overestimates cases, and biomarker confirmation appears to be a necessary part of the diagnostic paradigm. Use of plasma thiamine to confirm diagnosis of TD identified cases which seemed to improve after thiamine replenishment, indicating that it is a potentially useful biomarker. Whole-blood thiamine deficiency was infrequent and appeared to miss many cases of TRDs. WE in those without AUD primarily manifests as altered mental status with a minority having ophthalmoplegia or ataxia.

## Figures and Tables

**Table 1:** Biomarker thiamine deficiency by demographics for 263 participants.

	Low Plasma Thiamine ^¥^ (n = 69)		Low Whole-Blood Thiamine ^€^ (n = 7)	
	n (%)	*p*-Value	n (%)	*p*-Value
Age				
<50 (N = 15)	6 (8.70%)	0.1240	0 (0.00%)	0.0340
50–59 (N = 15)	5 (7.25%)		0 (0.00%)	
60–69 (N = 62)	21 (30.43%)		4 (57.14%)	
70–79 (N = 120)	29 (42.03%)		2 (28.57%)	
≥80 (N = 51)	8 (11.59%)		1 (14.29%)	
Unknown (N = 0)	0 (0.00%)		0 (0.00%)	
Birth Sex				
Male (N = 252)	64 (92.75%)	0.1658	5 (71.43%)	0.0255
Female (N = 11)	5 (7.25%)		2 (28.57%)	
Unknown (N = 0)	0 (0.00%)		0 (0.00%)	
Ethnicity				
Non-Hispanic (N = 249)	67 (97.10%)	1.0000	7 (100.00%)	1.0000
Hispanic (N = 9)	2 (2.90%)		0 (0.00%)	
Unknown (N = 5)	0 (0.00%)		0 (0.00%)	
Race				
White (N = 227)	62 (89.86%)	0.6181	5 (71.43%)	0.1110
African American (N = 11)	4 (5.80%)		1 (14.29%)	
Asian (N = 2)	0 (0.00%)		0 (0.00%)	
AIAN (N = 6)	0 (0.00%)		0 (0.00%)	
NHPI (N = 3)	0 (0.00%)		0 (0.00%)	
Unknown (N = 14)	3 (4.35%)		1 (14.29%)	

N is number of participants per row category; n is the number of participants with deficiency per column category. ¥ is less than 8 nmol L^−1^. € is less than 66.5 nmol L^−1^. Participants with missing demographic information were retained in descriptive summaries as “Unknown” but excluded from statistical analyses. Fisher’s exact test tested associations between demographic features and low plasma thiamine/whole-blood thiamine (low vs. normal) because 25% of expected cell frequencies < 5. *p*-values < 0.05 are considered significant. AIAN = American Indian/Alaskan Native; NHPI = Native Hawaiian/Pacific Islander.

**Table 2:** Baseline characteristics of participants who did versus did not complete a follow-up clinical assessment.

	Follow-Up (n = 60)	No Follow-Up (n = 123)	*p*-Value
Average Age (years)	69 ± 10	72 ± 12	0.2000
Age Group			
<50 (N = 11)	5 (8.33%)	6 (4.88%)	0.0456
50–59 (N = 47)	2 (3.33%)	9 (7.32%)	
60–69 (N = 83)	19 (31.67%)	28 (22.76%)	
70–79 (N = 11)	30 (50.00%)	53 (43.09%)	
≥80 (N = 31)	4 (6.67%)	27 (21.95%)	
Birth Sex			
Male (N = 175)	57 (95.00%)	118 (96.00%)	0.7000
Female (N = 8)	3 (5.00%)	5 (4.1%)	
Average Plasma Thiamine	9 ± 4	16 ± 16	<0.0010
Plasma Thiamine			
Deficient ^¥^ (N = 49)	32 (53.33%)	17 (13.82%)	<0.0010
Not Deficient (N = 132)	28 (46.67%)	104 (84.55%)	
Unknown (N = 2)	0 (0.00%)	2 (1.63%)	
Symptom Count	4.28 ± 2.19	3.50 ± 2.14	0.0140

Continuous variables are presented as mean ± standard deviation (SD); categorical variables are presented as n (%). ¥ plasma thiamine less than 8 nmol L^−1^. N is the total number of participants within each row category. *p*-values were calculated using the Wilcoxon rank-sum test for continuous variables and Fisher’s exact test for categorical variables. Participants with missing values for a given variable were excluded from the corresponding statistical analysis. *p*-values ≤ 0.05 were considered statistically significant.

**Table 3:** Comparison of clinical scores before and after thiamine repletion for 32 participants with low plasma thiamine (<8 nmol L^−1^).

	Mean Change in Score	95% C.I.	*p*-Value
MOCA test score	+1.9 points	[0.505, 3.295]	0.0093
Motor strength composite score	+2.5	[3.900 × 10^−5^, 5.000]	0.0417
Extraocular movement composite score	+2.5	[−1.000, 4.500]	0.1419
Ataxia composite score	+1.41	¥	0.1736

MOCA is Montreal Cognitive Assessment. C.I. is confidence interval.. Extraocular movement scores were 0 for disconjugate/nystagmus and 1 for conjugate, so an increase in score indicates improvement. Ataxia scores were 0 for ataxia and 1 for no ataxia, so an increase in score indicates improvement. *p*-values ≤ 0.05 are considered significant. Whole-blood biomarker deficiency is not included due to the low number of abnormal results. ¥ designates inability to compute 95% C.I. due to limited variability in the ataxia scores with many identical values.

**Table 4:** Associations among low plasma thiamine (<8 nmol L^−1^), low-whole-blood thiamine (<66.5 nmol L^−1^), and clinical indicators of medical comorbidity for n = 263 participants.

	Low Plasma Thiamine ^¥^ (n = 69)				Low Whole-Blood Thiamine ^€^ (n = 7)			
	n (%)	*p*-Value	IRR/Odds Ratio	95% C.I.	n (%)	*p*-Value	IRR/Odds Ratio	95% C.I.
Readmission from prior 30-day hospitalization								
Yes (N = 37)	6 (8.70%)	0.1281	0.495	[0.179, 1.167]	1 (14.29%)	1.0000	1.033	[0.054, 6.302]
No (N = 226)	63 (91.30%)		Ref	Ref	6 (85.71%)		Ref	Ref
Unknown (N = 0)	0 (0.00%)		--	--	0 (0.00%)		--	--
Readmitted within 30 days of discharge from enrollment hospitalization								
Yes (N = 47)	12 (17.39%)	0.9527	0.978	[0.459, 1.975]	1 (14.29%)	1.0000	0.767	[0.040, 4.640]
No (N = 216)	57 (82.61%)		Ref	Ref	6 (85.71%)		Ref	Ref
Unknown (N = 0)	0 (0.00%)		--	--	0 (0.00%)		--	--
Death within 90 days								
Yes (N = 31)	7 (10.14%)	0.7086	0.843	[0.321, 1.974]	1 (14.29%)	0.5964	1.228	[0.064, 7.532]
No (N = 231)	61 (88.41%)		Ref	Ref	6 (85.71%)		Ref	Ref
Unknown (N = 1)	1 (1.45%)		--	--	0 (0.00%)		--	--
Hospital length of stay (days)								
	--	0.4738	1.034	[0.836, 1.283]	--	0.3402	1.178	[0.669, 2.166]

N is number of participants per row category; n is the number of participants with deficiency per column category. ¥ is less than 8 nmol L^−1^. € is less than 66.5 nmol L^−1^. IRR is incidence rate ratio. C.I. = confidence interval. Reference groups (Ref) for each were defined as cases in which the event did not happen. Participants with missing outcome data were retained in descriptive summaries as “Unknown” but excluded from statistical analyses. Chi-square and logistic regressions tested associations between binary clinical indicators of medical comorbidity and low plasma and whole-blood thiamine (low vs. normal). Wilcoxon rank-sum and negative binomial regression tested associations between continuous indicators of medical comorbidity (e.g., LOS) and low plasma and whole-blood thiamine. *p*-values < 0.05 were considered significant. Double dash (--) indicates that the statistic was not calculated because the category contained no participants or because an effect estimate could not be estimated.

## Data Availability

Data that support the findings of this study are available upon request from the corresponding author. The data are not publicly available due to privacy or ethical restrictions.

## Abbreviations

The following abbreviations are used in this manuscript:

α-KGDHC	α-ketoglutarate dehydrogenase complex
AIAN	American Indian/Alaskan Native
AUD	alcohol use disorder
BCKDH	branched chain α-ketoacid dehydrogenase complex
BMI	body mass index
C.I.	confidence interval
ETKA	erythrocyte transketolase activation assay
GI	gastrointestinal
GLIM	Global Leadership Initiative on Malnutrition
HPLC	high-performance liquid chromatography
IRR	incidence rate ratio
MS	mass spectroscopy
LC-MS	liquid chromatography/mass spectroscopy
LOS	hospital length of stay
mg	milligrams
mg dL^−1^	milligrams per deciliter
MOCA	Montreal cognitive assessment
NHPI	Native Hawaiian/Pacific Islander
nmol L^−1^	nanomoles per liter
OR	odds ratio
PDC	pyruvate dehydrogenase complex
RBC	red blood cell
T+	free thiamine
TD	thiamine deficiency
TDDs	thiamine deficiency disorders
TDP	thiamine diphosphate
TMP	thiamine monophosphate
TRDs	thiamine-responsive disorders
US	United States
VA	Veterans Administration
VASNHCS	VA Sierra Nevada Healthcare System
VINCI	Veterans Affairs Informatics and Computing Infrastructure
WE	Wernicke’s encephalopathy

## Appendix A

Questions to assess participant comprehension of informed consent elements [30]

What is your understanding of what you will be doing?

What are the risks of participating in this research?

What are the benefits of participating in this research study?

How did you make your decision to participate in the study?

For each of the above questions, interviewer rates them as either “able to express” or “unable to express” the answer. If they are unable to express any of the answers, they were not enrolled.

Do you wish to participate in this study?

Do you have to participate?

For these questions, answers are “yes” or “no”. If they do not want to participate or answer “yes” to the question “do you have to participate in this study”, they were not enrolled.

## Appendix B

Symptom and function interview questions for participants

Individuals were asked “Have you recently (<3 months) noticed increased trouble with any of the following:“

Weakness or falls

Double vision

Confusion or trouble thinking

Memory loss

Hallucinations (seeing or hearing things that aren’t there)

Seizures

Nausea or vomiting

Constipation

Loss of balance

Abnormal sensation in hands, feet, or legs (burning, cold, pain, no feeling at all).

They were also asked

Have you eaten less than usual the past 7 days?

Is your appetite decreased?

Have you lost weight without trying?

For each question respondent was asked to answer “yes”, “no”, or “I don’t know”.
